# Changes in haematopoietic progenitor colony differentiation and proliferation and the production of different abzymes in EAE mice treated with DNA

**DOI:** 10.1111/jcmm.13289

**Published:** 2017-08-05

**Authors:** Kseniya S. Aulova, Ludmila B. Toporkova, Julia A. Lopatnikova, Alina A. Alshevskaya, Sergei V. Sennikov, Valentina N. Buneva, Thomas Budde, Sven G. Meuth, Nelly A. Popova, Irina A. Orlovskaya, Georgy A. Nevinsky

**Affiliations:** ^1^ Institute of Chemical Biology and Fundamental Medicine Siberian Branch of the Russian Academy of Sciences Novosibirsk Russia; ^2^ Institute of Clinical Immunology Siberian Branch of the Russian Academy of Sciences Novosibirsk Russia; ^3^ Westfälische Wilhelms‐Universität Institut für Physiologie I Münster Germany; ^4^ Department of Neurology Westfälische Wilhelms‐Universität Münster Germany; ^5^ Institute Cytology and Genetics Siberian Branch of the Russian Academy of Sciences Novosibirsk Russia; ^6^ Novosibirsk state university Novosibirsk Russia

**Keywords:** EAE model, C57BL/6 mice, immunization with DNA, catalytic antibodies, colony formation, haematopoietic progenitor differentiation

## Abstract

Immunization of experimental autoimmune encephalomyelitis (EAE)‐prone C57BL/6 mice with MOG_35‐55_ (a model used to study aspects of human multiple sclerosis) is known to lead to the production of various abzymes. The production of catalytic IgGs that can efficiently hydrolyse myelin basic protein (MBP), MOG and DNA is associated with changes in the profile of differentiation and level of proliferation of mice bone marrow haematopoietic stem cells (HSCs). As MOG simulates the production of abzymes with high DNase activity, we compared the effects of DNA and MOG immunization on EAE‐prone mice. In contrast to MOG, immunization with DNA leads to a suppression of proteinuria, a decrease in the concentrations of antibodies to MOG and DNA and a reduction in abzyme production. Immunization with DNA only resulted in a significant increase in DNase activity over 40 days where it became 122‐fold higher than before immunization, and fivefold higher when comparing to the maximal activity obtained after MOG treatment. DNA and MOG immunization had different effects on the differentiation profiles of HSCs, lymphocyte proliferation, and the level of apoptosis in bone marrow and other organs of mice. The data indicate that for C57BL/6 mice, DNA may have antagonistic effects with respect to MOG immunization. The usually fast immune response following MOG injection in C57BL/6 mice is strongly delayed after immunization with DNA, which is probably due to a rearrangement of the immune system following the response to DNA.

## Introduction

Multiple sclerosis (MS) is characterized by inflammatory and demyelinating conditions in the central nervous system (CNS) as well as perivascular infiltrates composed largely of T lymphocytes and macrophages. Numerous avenues of research support the role of autoimmune mechanisms in the destruction of myelin, although the precise cause of MS remains unknown 1. Data show that activated CD4^+^ myelin‐reactive T cells may be major mediators of MS 1. Several recent results specify an important role of B cells and autoantibodies (auto‐Abs) against myelin autoantigens in the pathogenesis of MS 1, 2, 3.

An important and potential dual role of autoantibodies in MS has been previously proposed: auto‐Abs may be harmful in lesion formation, or potentially beneficial in lesion repair 2. Increased levels of Abs and oligoclonal IgGs in the cerebrospinal fluid (CSF), accumulation of clonal B cells in the CSF and lesions of MS patients are among the main lines of evidence for the involvement of a humoural response in demyelination 4. Current evidence from clinical studies and animal models suggests that auto‐Abs against myelin components (involved in antibody‐mediated demyelination 3), as well as auto‐Abs against oligodendrocyte progenitor cell protein, which could impair remyelination by eliminating or impeding these cells 5, may play a crucial role in MS immunopathogenesis.

Artificial antibodies against transition states of different chemical reactions and natural abzymes present in the blood of autoimmune patients are involved in catalysing more than 200 distinct chemical reactions and are novel biologic catalysts that have attracted a lot of interest over recent years (for review, see 6, 7, 8, 9).

In the sera of healthy human beings and animals, it is possible to detect auto‐Abs against different antigens including DNA and various proteins, even though their titres vary significantly and they are usually catalytically inactive 9, 10, 11, 12, 13, 14, 15, 16. The presence of natural abzymes that hydrolyse various oligopeptides, proteins, nucleic acids and polysaccharides has been revealed in the sera of patients with many different autoimmune diseases (for review, see 9, 10, 11, 12, 13, 14). Interestingly, the catalytic activity of DNase and (MBP)‐hydrolysing abzymes is easily detectable during the onset of these diseases. Detection is even possible when titres of Abs against DNA, MBP or other autoantigens have not yet significantly increased and correspond to levels found in healthy donors 10, 11, 12, 13, 14. It was shown that IgGs from the sera and cerebrospinal fluid (CSF) of MS patients are active in the hydrolysis of MBP, DNA and polysaccharides 17, 18, 19, 20, 21, 22, 23, 24, 25, 26, 27, 28. In addition, the relative activities (RAs) of these abzymes from the CSF of MS patients are approximately from 30‐ to 60‐fold higher on average than those from sera of the same patients 25, 26, 28. In MS, abzymes with MBP‐hydrolysing activities can attack MBP in the myelin‐proteolipid sheath of axons. The established MS drug Copaxone^®^ (glatiramer acetate) was shown to be a specific inhibitor of MBP‐hydrolysing abzyme activity 27. Therefore, these abzymes may play an important and harmful role in MS pathogenesis.

Abzymes with different activities were revealed with a dramatically higher incidence in autoimmune mouse lines than in conventionally used control non‐autoimmune mouse strains 29, 30. Anti‐DNA Abs in systemic lupus erythematosus (SLE) are usually directed against histone–DNA nucleosomal complexes, appearing as a result of internucleosomal cleavage during apoptosis 31.

It has been suggested that autoimmune diseases originate from defects in (HSCs) 32. In addition, it was shown for the first time that the specific reorganization of the immune system during the spontaneous and DNA‐induced development of a profound SLE‐like pathology in MRL‐lpr/lpr mice is associated with changes in the differentiation profile of bone marrow HSCs in combination with the production of DNase, ATPase and polysaccharide‐hydrolysing abzymes 33, 34, 35. Immunization of non‐autoimmune CBA and BALB/c healthy mice with DNA also leads to the production of Abs with very low DNase activity; however, this is only associated with increased lymphocyte proliferation and suppression of lymphocyte apoptosis in different organs (especially the spleen), and not with marked changes in the differentiation of bone marrow cells 35.

Several different EAE models are available, each mimicking a particular facet of MS (for review, see 36). We have recently used C57BL/6 mice to study the possible mechanisms of spontaneous and MOG‐accelerated development of EAE 37. It was shown that specific reorganization of the immune system occurred during development of spontaneous and MOG‐induced EAE, leading to a condition that was associated with the generation of catalytically active IgGs hydrolysing MOG, MBP and DNA. However, treating mice with MOG results in very strong acceleration in the development of EAE, and a very strong increase in the relative activities of abzymes hydrolysing MOG, MBP and DNA 37. An especially unexpected finding was that spontaneous and MOG‐induced EAE development led not only to production of abzymes hydrolysing MOG and MBP, but also to Abs that efficiently hydrolyse DNA 37, such as in the case of MRL‐lpr/lpr mice immunized with DNA 33, 34, 35. Therefore, here we treated C57BL/6 mice with DNA and analysed biochemical markers of EAE pathology (proteinuria, Ab titres to MOG, MBP and DNA) as well as RAs of mouse IgGs in the hydrolysis of MBP, MOG and DNA. In addition, changes in the profile and level of proliferation of bone marrow HSCs before and after treatment of mice with DNA were analysed. The changes in these parameters after treatment of mice with DNA were compared with those for spontaneous and MOG‐induced EAE.

## Materials and methods

### Reagents

Most reagents including chemicals, proteins, methylated bovine serum albumin (m‐BSA), polymeric bovine DNA, Protein G Sepharose and the Superdex 200 HR 10/30 column were purchased from Sigma‐Aldrich (Munich, Germany) or GE Healthcare. Human MBP of 18.5 kD was obtained from the Research Centre of Molecular Diagnostics and Therapy (Moscow, Russia), and MOG_35‐55_ was obtained from EZBiolab. These preparations were free from lipids, oligosaccharides, nucleic acids and other possible contaminants.

### Experimental animals

Inbred 3‐month‐old C57BL/6 mice were housed in colonies under standard pathogen‐free conditions with a system for protection from bacterial and viral infections at the Institute of Cytology and Genetics mouse breeding facility. All experimental procedures with mice were performed according to protocols from the Bioethical Committee of the Institute of Cytology and Genetics. These correspond to protocols from the Bioethical Committee of the Siberian Branch of the Russian Academy of Sciences and the recommendations of the European Committee for the humane principles of work with experimental animals (European Communities Council Directive 86/609/CEE). The Bioethical Committee of the Institute of Cytology and Genetics has approved our study in accordance with the guidelines of the European Communities Council Directive 86/609.

Experimental autoimmune encephalomyelitis mice were immunized twice with 40 μg of polymeric thymus DNA that was conjugated with methylated BSA and dissolved in physiological solution as described previously 35, 38. A mixture of 0.5 volume of complete Freund's adjuvant and 0.5 volume of antigen solution was used. The mixture was stirred to form a homogeneous gel and was injected subcutaneously or into paw pads. The second immunization with a mixture containing incomplete Freund's adjuvant was performed after 2 days. Immunization of mice with MOG_35‐55_ and pertussis toxin (*Mycobacterium tuberculosis)* was conducted as described in 37 according to 41. The relative weight of mice and proteinuria (relative concentration of total protein in the urine, mg/ml) were analysed as before 35. Protein concentration in urine was measured using the Bradford assay with a bovine serum albumin standard. For different experiments including purification of Abs and analysis of their enzymatic activity, 0.7–1 ml of blood was collected after decapitation using standard approaches 37.

### ELISA of antiprotein and anti‐DNA Abs

Anti‐MOG and anti‐MBP antibody concentrations were measured by ELISA (plasma was diluted 50‐fold) according to 37. The concentration of anti‐DNA Abs was estimated using standard ELISA plates with immobilized double‐stranded DNA (plasma was diluted 100‐fold) as described previously 35, 37. After a consecutive treatment of blood plasma samples with rabbit‐specific anti‐mouse Abs conjugated with horseradish peroxidase, the analysed mixtures were incubated with tetramethylbenzidine and H_2_O_2_. The reaction was stopped with H_2_SO_4_, and the optical density (A_450_) of the solutions was determined using a Uniskan II plate reader MTX Lab Systems, (Bradenton, USA). The relative concentrations of anti‐MOG, anti‐MBP and anti‐DNA antibodies in the samples were expressed as a difference in the relative absorption at 450 nm between experimental and control samples; controls with MOG, MBP or DNA, but without serum samples and with Abs not interacting with MOG, MBP or DNA, gave the same results.

### IgG purification

Electrophoretically and immunologically homogeneous mouse IgGs were purified using sequential chromatography of plasma proteins on Protein G Sepharose following FPLC gel filtration as described previously 22, 23, 24, 33, 34, 35. For protection of Abs from bacterial and viral contamination, they were filtered through Millex syringe‐driven filter units (0.2 μm) and kept in sterilized tubes. Ab preparations were checked for the absence of bacterial and viral contamination as in 37. SDS‐PAGE analysis of the IgGs for homogeneity was performed using 4–15% gradient gels and non‐reducing conditions; for polypeptide separation, a reducing gel (0.1% SDS and 10 mM dithiothreitol) was used. Intact IgGs and their heavy and light chains were visualized by silver staining as in 22, 23, 24, 33, 34, 35, 37. To exclude possible contamination by canonical enzymes, IgGs were separated by SDS‐PAGE and their protease and nuclease activities were detected using a gel assay as before [22, 23, 24, 33, 34, 35, 37

### DNA‐hydrolysing activity assay

DNase activity was analysed as described in 35, 37, 38. The reaction mixture (20 μl) containing 20 μg/ml supercoiled (sc) pBluescript, 20 mM NaCl, 5 mM MgCl_2_, 1 mM EDTA, 20 mM Tris–HCl (pH 7.5) and 0.03–0.2 mg/ml of Abs was incubated for 1–12 hrs at 37^o^ C. Products of DNA hydrolysis were separated by electrophoresis on 0.8% agarose gels. Ethidium bromide‐stained gels were photographed and analysed using Gel‐Pro Analyzer v9.11. The relative catalytic activity was calculated from the percentage of DNA corresponding to an initial band of scDNA and its relaxed form, and a distribution of DNA between these bands in the case of the control experiment (incubation of plasmid in the absence of Abs) was taken into account. All initial rates of the reactions were analysed within the linear regions of the time course (15–40% of DNA hydrolysis), and a complete transition of scDNA to nicked DNA was taken as 100% of the activity. If the activity was low (<5–10% of scDNA disappearance), the incubation time was increased to 3–12 hrs, depending on the sample. If the degradation of scDNA exceeded 50%, the concentration of Abs was decreased two‐ to 100‐fold, depending on the sample analysed. Finally, the RAs were normalized to the same conditions.

### Protease activity assay

The reaction mixtures (10–40 μl) for analysis of MBP‐ or MOG‐hydrolysing activities of IgGs contained 20 mM Tris‐HCl (pH 7.5), 0.7–1.0 mg/ml MBP or MOG and 0.01–0.2 mg/ml of IgGs. The mixtures were incubated for 1–20 hrs at 37°C. The cleavage products of MBP and MOG were analysed by SDS‐PAGE on 12% or 4–15% gradient gels with Coomassie R250 staining. The gels were scanned and quantified using GelPro v3.1 software. The RAs of different IgGs were determined as a decrease in the percentage of initial MBP or MOG converted to different hydrolysed forms compared with control MBP or MOG incubated without Abs. All measurements (initial rates) were taken under the conditions of the pseudo‐first order of the reaction within the linear regions of the time course (15–40% of MBP or MOG) and dependencies of the substrates hydrolysis on IgG concentration.

### Analysis of bone marrow progenitor cells in culture

Bone marrow was flushed out from mouse femurs, and the colony‐forming ability of bone marrow cells was assessed. 2 × 10^4^ cells (four dishes per mouse) were cultured in a standard methylcellulose‐based M3434 medium for mouse cells Stemcell Technologies (Cambridge, England). The medium contained stem cell factor, erythropoietin (EPO), interleukin (IL)‐3 and IL‐6. BFU‐E, CFU‐GM, CFU‐E and CFU‐GEMM colonies were scored after 14 days of incubation at 37°C and 5% CO_2_ in a humidified incubator as described previously 33, 34, 35, 37.

### Analysis of lymphocyte proliferation

*In vitro* analysis of lymphocyte proliferation (sum of T and B cells) was carried out as in 37. Cells (10^6^/ml) were isolated from bone marrow, thymus, lymph nodes and spleen and then cultivated in 96‐well flat‐bottom plates (Trasadingen, Switzerland) with RPMI‐1640 medium containing 10% of foetal calf serum, 10 mM HEPES buffer, 0.5 mM 2‐mercaptoethanol, 2 mM l‐glutamine, 100 μg/ml benzylpenicillin and 80 μg/ml gentamicin. After incubation for 64 hrs, 15 μl solution containing 5 mg/ml MTT (tetrazolium dye MTT 3‐(4,5‐dimethylthiazol‐2‐yl)‐2,5‐diphenyltetrazolium bromide) was added to every well; the plates were incubated for at 37°C for 4 hrs. Then, plates were centrifuged for 10 min at 1200 × *g* and solutions were removed. Cells were precipitated by the addition of DMSO (200 μl), resuspended and incubated at room temperature for 15 min in darkness. The analysis of cells was performed spectrophotometrically at 492 nm.

### Apoptosis assay

Analysis of apoptosis was carried out according to 37. Cells (1 × 10^6^/ml) were incubated at 37°C for 48 hrs in RPMI‐1640 medium containing foetal calf serum (10%), 10 mM HEPES buffer, 0.5 mM 2‐mercaptoethanol, 2 mM l‐glutamine, 100 μg/ml benzylpenicillin and 80 μg/ml gentamicin (5% CO_2_). Then, cells were washed using 2 ml phosphate‐buffered saline containing 0.1% NaN_3_ and 0.02% EDTA 37. The cells were incubated with 0.5 ml solution containing 250 μg/ml RNase, 0.1% Triton X‐100 and 50 μg/ml propidium iodide (PI) at 37°C for 20 min. PI‐fluorescence was determined using a BD FACSVerse flow cytometer at the level of individual nuclei. The results are given as the percentage of fragmented (hypodiploid) nuclei reflecting the fraction of apoptotic cells.

### Statistical analysis

The results are reported as the mean ± S.D. of at least three to four independent experiments for each animal, averaged over seven different mice. Differences between the samples and mouse groups were analysed by Student's *t*‐test; *P* ≤ 0.05 was considered to be statistically significant.

## Results

### Experimental groups of mice

In this study, we have used three experimental groups of C57BL/6 mice (seven animals for each time‐point):

1. untreated control C57BL/6 mice,
2. DNA‐treated C57BL/6 mice,
3. MOG‐immunized C57BL/6 mice.

C57BL/6 mice were immunized with conjugates of polymeric DNA and methylated BSA (hereinafter referred to as ‘DNA’) as performed previously for the treatment of autoimmune MRL‐lpr/lpr mice 35. Usually, anti‐DNA Abs in many autoimmune diseases are directed against DNA–histone nucleosomal complexes; these complexes appear as a result of internucleosomal cleavage during apoptosis 31. Positively charged methylated BSA was used to simulate positively charged histones forming strong complexes with DNA.

**Figure 1:**
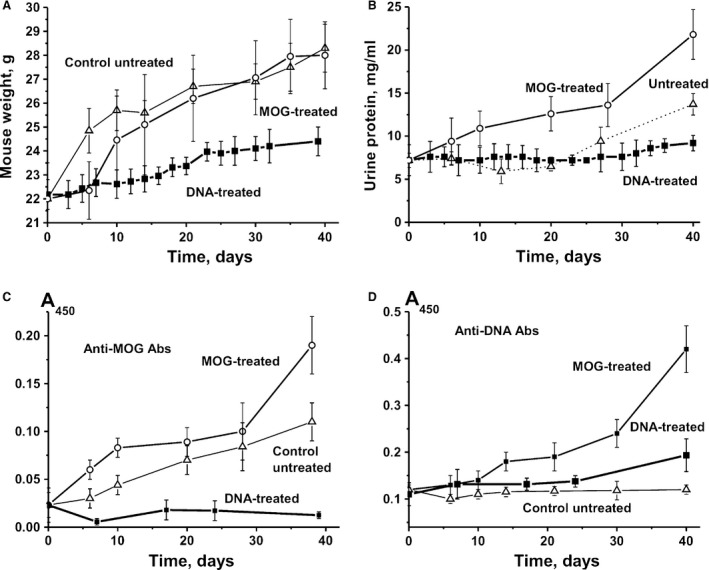
Relative changes in parameters characterizing C57BL/6 EAE mice over time. Shown are changes in bodyweight (**A**)**,** proteinuria (**B**), titres of Abs to DNA (**C**) and to MOG (**D**) for groups of untreated control mice, and those treated with DNA and MOG. Concentrations of anti‐MOG and anti‐double‐stranded DNA in the sera of C57B2/6 mice were measured using a standard ELISA approach where plasma was diluted 50‐ and 100‐fold, respectively; A_450_—the absorbance at 450 nm.

Possible changes in the relative weight along with a number of immunological and biochemical parameters were analysed in DNA‐treated C57BL/6 mice at 3 months of age (zero time‐point; control) and for 40 consecutive days. Control experiments with MOG‐treated mice were performed in parallel to compare the effects of DNA and MOG treatment. These control experiments were performed similarly to 37; the data obtained here were not significantly different from previously published results indicating that the conditions were highly reproducible. Treatment of C57BL/6 mice with MOG leads to reduced weight gain during the first seven days compared to untreated mice, although the average weight increased gradually to approximately match the weight of control mice at day 40 (Fig. 1A). Unexpectedly, immunization of mice with DNA prevented the increase in weight over time, and at day 40, it was significantly lower (~1.2‐fold; *R* < 0.05) than for MOG‐treated and untreated mice (Fig. 1A).

In MRL‐lpr/lpr and other animal autoimmune models, the appearance of visible symptoms usually correlates well with high levels of proteinuria (≥3 mg/ml concentration of protein in urine) 33, 34, 35, 36. Control non‐autoimmune BALB and CBA mice do not demonstrate proteinuria for an observation period of at least 7 months (0.1‐0.12 mg/ml) 33, 34, 35, 36. Healthy MRL‐lpr/lpr mice exhibit low proteinuria (0.38 mg/ml) before development of spontaneous or DNA‐induced pronounced SLE 33, 34, 35, 36. In MOG‐induced C57BL/6 mice, development of the first clinical symptoms is usually observed at 5–7 days after immunization, while the maximum stage of the disease usually manifests at 14‐20 days after treatment 39, 40. At the same time, C57BL/6 mice display a significantly higher level of proteinuria (up to 10–12 mg/ml) at 3 months of age, even before immunization with MOG 41, 42. We analysed the time‐dependent change in proteinuria for control, DNA‐ and MOG‐treated C57BL/6 mice (Fig. 1B). In all groups, the average urine protein concentration was 7.2 ± 0.8 mg/ml at time‐point zero (3 months of age). While proteinuria gradually increased over time to 21.8 mg/ml in MOG‐treated mice, it remained nearly constant in untreated control mice until day 20 and then increased to 12.0 mg/ml by day 40. However, in the case of mice treated with DNA, levels of proteinuria did not exhibit a marked change over time. Urine protein levels were 2.4‐ and 1.5‐fold lower compared to MOG‐treated and untreated mice, respectively (Fig. 1B). Thus, treatment of mice with DNA leads not only to a statistically significant (*P* < 0.05) decrease in weight, but also the proteinuria in comparison with untreated and MOG‐treated EAE mice did not increase but remained nearly constant.

### Analysis of the relative content of anti‐DNA and anti‐MOG Abs

It is known that sera of healthy human beings and sera of MS and SLE patients usually contain anti‐DNA 17, 18 and anti‐MBP 22, 23, 24 Abs. The relative concentration of anti‐DNA Abs of non‐autoimmune BALB and CBA mice at 2–7 months of age and healthy MRL‐lpr/lpr mice (2–3 months of age) is usually low and varies in the range of 0.03–0.04 A_450_ units 35. Using the same ELISA test, we have estimated the relative concentration of anti‐DNA Abs over time in the blood of C57BL/6 mice (Fig. 1C). At 3 months of age, the average concentration of anti‐DNA Abs in untreated C57BL/6 mice was ~0.12 A_450_ units, and it remained at a similar level throughout 40 days of analysis (Fig. 1C). Interestingly, the treatment of mice with DNA led to a slow increase in the concentration of anti‐DNA Abs up to 25 days, but at 40 days, it became 1.7‐fold higher (*P* < 0.05) than at the beginning. The relative concentration of anti‐DNA Abs in the case of MOG‐treated mice gradually increased and at 40 days was 3.8‐fold higher (*P* < 0.05) than that at time‐point zero (Fig. 1C). Surprisingly, treatment of mice with DNA leads to a significantly lower increase in anti‐DNA Abs at day 40 compared with immunization with MOG (Fig. 1C).

Figure 1D demonstrates time‐dependent changes in anti‐MOG Abs in the sera of untreated, DNA‐ and MOG‐treated C57BL/6 mice. The average relative concentration of anti‐MOG antibodies of spontaneously diseased untreated mice increased 4.8‐fold (*P* ≤ 0.05) during ~40 days in a nearly linear fashion. Treatment of mice with MOG accelerated the increase in concentration of anti‐MOG antibodies over 6‐10 days; then, at day 40, it increased to 8.3‐fold higher than at time‐point zero (Fig. 1D). Immunization of mice with DNA led to an almost complete suppression of the production of anti‐MOG antibodies at 7 days after immunization, while at 40 days, it was twofold lower than at the zero time‐point and nine‐ and ~16‐fold lower when compared to untreated and MOG‐treated mice. Thus, immunization of mice with DNA slows their growth and has only a small effect on proteinuria, but greatly suppresses the production of anti‐MOG antibodies, which was observed for untreated mice and MOG‐induced development of EAE. It seems that DNA prevents the stimulation of antibody production against DNA and MOG in C57BL/6 mice, while MOG stimulates the production of Abs to both MOG and DNA.

### Determination of the relative proteolytic and DNase activities of antibodies

**Figure 2:**
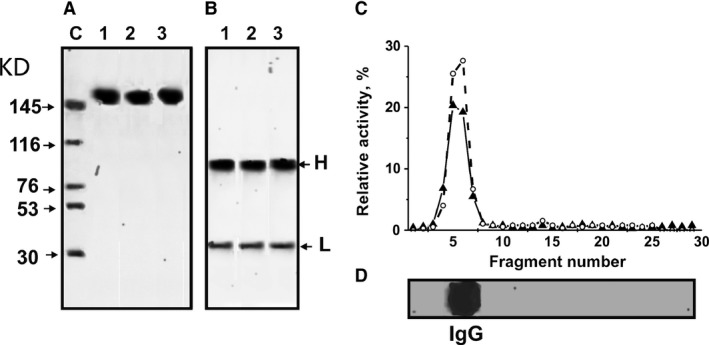
SDS‐PAGE analysis of the homogeneity of dna‐IgG_mix_ (lane 1), mog‐IgG_mix_ (lane 2) and untret‐IgG_mix_ (lane 3) (7 μg) under non‐reducing (**A**, lanes 1–3) and reducing (**B**, lanes 1–3) conditions followed by silver staining; the arrows (**A**, lane C) show the positions of protein markers with known molecular masses. The relative activities (RA, %) of proteins hydrolysing DNA (o) and MOG (▲) were revealed using the extracts of fragments (2–3 mm) of one longitudinal slice of the gel (**C**). Panel **D** shows IgG position of after the electrophoresis. A complete hydrolysis of these substrates during 24 hrs was taken for 100% (**C**). The error in the initial determination of RAs from two independent experiments did not exceed 7–10%. For other details, see Materials and methods.

To search for abzymes, IgGs were purified from the plasma of individual mice using chromatography on Protein G Sepharose under special conditions to remove non‐specifically bound proteins as previously described 22, 23, 24, 33, 34, 35. Then, IgGs were additionally purified by FPLC gel filtration. For analysis of proteolytic and DNase activities, we took IgGs from individual mice and created mixtures of equal amounts of electrophoretically homogeneous Abs from the plasma of untreated mice (untret‐IgG_mix_), MOG‐ (mog‐IgG_mix_) and DNA‐treated mice (dna‐IgG_mix_): three groups of seven mice at 40 days after treatment. The homogeneity of typical 150‐kD untret‐IgG_mix,_ mog‐IgG_mix_ and dna‐IgG_mix_ was analysed by SDS‐PAGE with silver staining. Samples showed a single band under non‐reducing conditions (*e.g*. Fig. 2A) and two bands corresponding to the heavy and light chains after Ab reduction (Fig. 2B). IgGs from control non‐autoimmune CBA and BALB mice (3–7 months of age) did not demonstrate detectable activity in the hydrolysis of DNA, MOG and MBP, while IgGs from untreated and MOG‐treated EAE mice showed high activity in hydrolysis of these substrates 33, 34, 35, 37. In this study, we confirmed previously obtained results; taking into account the errors in the estimation of activity values for seven MOG‐treated mice of each group, the average relative activity of Abs in this study was not different from that obtained in our previous studies 37. After treatment of C57BL/6 mice with DNA, IgGs were also active in the hydrolysis of DNA and MOG.

Based on strict criteria that were previously developed 10, 11, 12, 13, 14, we concluded that MOG‐hydrolysing and DNase activities are intrinsic properties of IgGs from the plasma of DNA‐treated C57BL/6 mice and are not due to co‐purified enzymes. This is due to a number of factors: (*i*) IgG preparations were shown to be electrophoretically homogeneous (Fig. 2A and B); (*ii*) FPLC gel filtration of the dna‐IgG_mix_ under conditions of acidic shock (pH 2.6) did not lead to a disappearance of these activities, and the peaks of protease and DNase enzymatic activities matched exactly with IgGs (data not shown); (*iii*) activities were completely absorbed by Sepharose‐bearing Abs against mouse IgG light chains, leading to a disappearance of the catalytic activities from the solution; activities were recovered with acidic buffer. To exclude possible artefacts due to trace amounts of contaminating canonical enzymes, the dna‐IgG_mix_ preparation was separated by SDS‐PAGE and the resulting gel lane was cut into 2‐ to 3‐mm‐sized fragments. Protease and DNA‐hydrolysing activities were then analysed after extraction of proteins from the separated gel slices (Fig. 2C and D). MOG‐ and DNA‐hydrolysing activities were revealed only in the fragments containing intact IgGs. SDS dissociates any protein complexes, and the electrophoretic mobility of canonical proteases and DNases (28‐32 kD) is significantly higher than that for intact IgGs (150 kD). Therefore, the detection of MOG‐hydrolysing and DNase activities in gel slices corresponding only to intact IgGs, along with the absence of any activity in the other gel fragments (Fig. 2), provides direct evidence that after treatment of C57BL/6 mice with DNA, the IgGs that are produced possess both activities.

**Figure 3:**
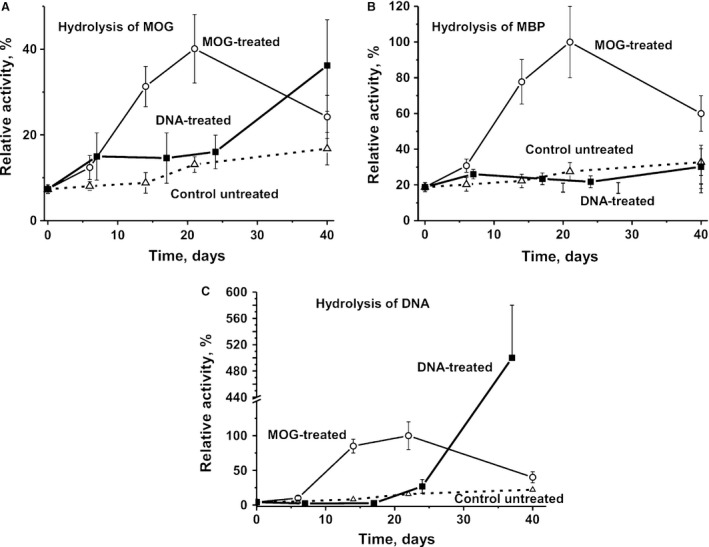
Analysis of relative MOG‐ (**A**) and MBP‐hydrolysing activities (**B**) of IgGs by SDS‐PAGE, and DNase activity by agarose gel electrophoresis. (**C**) The average values of RAs for groups of mice (each group of seven mice) in time after the treatment are given. The error in the initial rate determination from two experiments in the case of every mouse of each group did not exceed 7–10%. For other details, see Materials and methods.

We estimated time‐dependent changes in the average protease and DNase activities (RAs) of IgGs corresponding to individual Abs of the three experimental groups: untreated, MOG‐treated and DNA‐treated mice (each group of seven animals). Figure 3 demonstrates the results of the RAs of IgGs in the hydrolysis of MOG (A), MBP (B) and DNA (C). To quantify the MOG‐, MBP‐ and DNA‐hydrolysing activities of IgGs of different mice, we estimated a concentration for each individual IgG preparation that converted MOG and MBP to their hydrolytic products (no more than 40%) and scDNA to relaxed plasmid DNA without formation of linear or fragmented DNA (no more than 40% of initial DNA). As all measurements (initial rates) were taken within the linear regions of the time courses and Ab concentration curves, the measured RAs for IgGs were normalized to standard conditions. The data on changes in average activities for each group of seven mice over time are summarized in Figure 3.

During 40 days of the experiment, non‐treated control mice showed a nearly linear statistically significant increase in average MOG‐hydrolysing (1.8‐fold), MBP‐hydrolysing (1.7‐fold) and DNase (5.4‐fold) activities (Fig. 3). Immunization of mice with MOG led to a 5.4‐fold statistically significant increase in MOG‐hydrolysing activity at day 20. This time range corresponds to the acute stages of EAE disease, which usually manifest at 14–20 days after treatment 39, 40. At the 40‐day time‐point, which corresponds to the chronic stage of the disease, MOG‐hydrolysing activity was significantly decreased (Fig. 3A). The average activity of IgGs in MOG hydrolysis after treating mice with DNA was increased at 6–7 days corresponding to the time of the first clinical EAE symptoms that are usually observed at 5–7 days after immunization 39, 40, but then there were no further changes in the activity from 7 to 24 days. Compared to MOG‐immunized mice (significant changes at 14–20 days), a significant increase in MOG‐hydrolysing activity of mice treated with DNA was observed at a later time‐point of 40 days (Fig. 3A).

The RA in the hydrolysis of MBP during spontaneous development of EAE increased constantly over time, while mice treatment with MOG results in significant increase in this enzymatic activity from 6–7 to 20 days, which is to some extent similar to that observed for hydrolysis of MOG (Fig. 3B). Both MOG‐ and MBP‐hydrolysing activities decreased significantly at day 40 of the experiment (Fig 3A and B).

The average RA of MBP hydrolysis after treating mice with DNA was slightly increased at 6–7 days, although there were no statistically significant changes from 7 to 24 days (Fig. 3B). A very slight increase in MBP‐hydrolysing activity after immunization with DNA was observed only at 40 days (Fig. 3B). Interestingly, the RA of MBP hydrolysis of DNA‐treated mice at 40 days was relatively low and comparable with that for MBP hydrolysis by IgGs of untreated C57BL/6 mice (Fig. 3B).

In control, untreated C57BL/6 mice, the DNase activity of IgGs gradually increased from approximately 4.1 to 22% (5.4‐fold) over 40 days (Fig. 3C). The treatment of mice with MOG resulted in the increase in this activity by 24.4‐fold at day 22, which corresponds the acute phase of the disease. The relative DNase activity of IgGs of DNA‐treated mice almost unchanged during approximately 18 days (Fig. 3C). At the late stage of EAE (40 days), the average RA of IgGs from DNA‐treated mice in the hydrolysis of DNA was 122‐fold higher than that at time‐point zero, and fivefold higher than the maximal activity of abzymes corresponding to mice immunized with MOG at 20 days (Fig. 3C). Thus, the treatment of C57BL/6 mice with DNA leads to a significant delay in the development of autoimmune processes, but to a significant increase in the production of abzymes hydrolysing DNA at later time‐points. Interestingly, the very strong increase in DNase activity (Fig. 3C) does not correlate with the relatively moderate increase in the total concentration of anti‐DNA antibodies at day 40 (Fig. 1D). However, as was shown by us earlier, the correlation coefficients (0.1–0.4) between the total concentration of anti‐DNA antibodies and their catalytic activities can be very low 9, 10, 11, 12, 13, 14. One can suppose that immunization of mice with DNA first leads to a strong suppression of the production of antibodies to different antigens including MBP, MOG and DNA. Then, at later time‐points, the immune system switches from the synthesis of Abs without activities to production of abzymes with mostly DNase activities.

### Colony formation of haematopoietic progenitors

**Figure 4:**
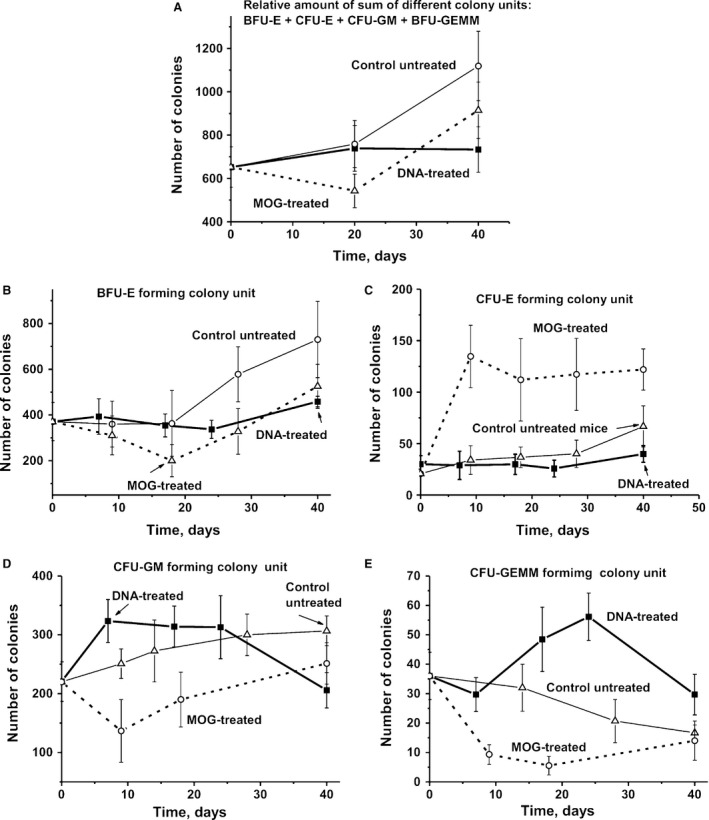
Changes in the total sum of all colonies (BFU‐E + CFU‐E + CFU‐GM + CFU‐GEMM) (**A**) as well as total number of specific colony units over time: BFU‐E (**B**), CFU‐E (**C**), CFU‐GM (**D**) and CFU‐GEMM (**E**) in the case of untreated, DNA‐ and MOG‐treated mice.

We first analysed changes in the sum of all colonies for BFU‐E (erythroid burst‐forming unit; early erythroid colonies), CFU‐E (erythroid burst‐forming unit; late erythroid colonies), CFU‐GM (granulocytic–macrophagic colony‐forming unit) and CFU‐GEMM (granulocytic, erythroid, myeloid colony‐forming unit) over time for untreated as well as DNA‐ and MOG‐treated mice (Fig. 4A). In untreated mice, the level of proliferation of the summed haematopoietic progenitor colonies initially increased slowly from 0 to 20 days and was followed by a statistically significant 1.7‐fold rise at day 40 in comparison with day zero (Fig. 4A). The treatment of mice with MOG leads to a 1.3‐fold (*P* < 0.05) decrease in the summed number of colonies at day 20 compared to day zero, but then increased at day 40 approximately by a factor of 1.4 in comparison with the initial level (Fig. 4A). Interestingly, the treatment of mice with DNA first leads to a slow increase in proliferation from 0 to 20 days at a similar rate as untreated mice, but then the number of colonies remains the same until day 40. However, the relative number of each type of individual colony‐forming unit in the case of untreated mice and after immunization with DNA or MOG is very different (Fig 4B and E).

In untreated control mice, the average total number of BFU‐E (~2.0‐fold), CFU‐GM (~1.4‐fold) and CFU‐E (~3.2‐fold) was significantly increased, while CFU‐GEMM (~2.2‐fold) gradually decreased over 40 days compared with the beginning of the experiment (Fig. 4). In MOG‐immunized mice, the number of BFU‐E (~1.9‐fold), CFU‐GM (~1.6‐fold) and CFU‐GEMM (~6.5‐fold) first decreased at 20 days after MOG injection and then markedly or significantly increased at 40 days (Fig. 4). In contrast to MOG‐treated and untreated mice, immunization of animals with DNA did not lead to a considerable change in the total number of BFU‐E and CFU‐E (Fig 4B and C). Interestingly, while following mice immunized with MOG, a significant decrease in CFU‐GM and CFU‐GEMM cells was observed, while DNA stimulated a significant increase in these colonies (Fig. 4D and E).

**Figure 5:**
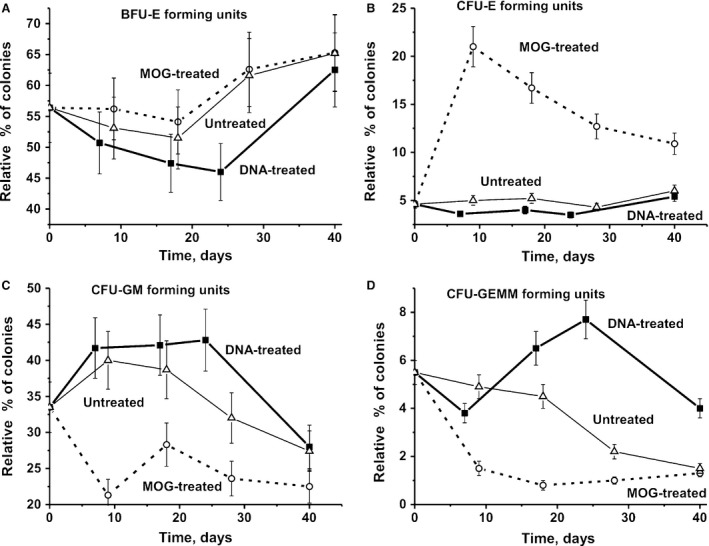
Changes in a relative per cent of BFU‐E (**A**), CFU‐E (**B**), CFU‐GM (**C**) and CFU‐GEMM (**D**) forming colony units for untreated, DNA‐ and MOG‐treated mice over time. The total number of all colonies was shown as 100%.

For an estimation of the relative amount of the four different types of colonies, we calculated the relative percentage of each colony type with respect to the total number of colonies. The relative percentage of BFU‐E in the case of MOG‐, DNA‐treated and untreated mice changed in a complex manner with a maximal decrease in the case of DNA‐treatment animals at 15–25 days (Fig. 5A). Interestingly, while the total number of CFU‐E colonies from DNA‐treated mice remains approximately the same from 0 to 40 days (Fig. 4B), the percentage content of BFU‐E after DNA treatment gradually decreases from 0 to 24 days, but then becomes higher at 40 days comparing to time‐point zero (Fig. 5A).

The total number of CFU‐E increased at 9 days after treatment of mice with MOG in comparison with day zero by a factor of 6.5, and then, there was no further significant change in cell numbers up to 40 days (Fig. 4C). Interestingly, in contrast to MOG‐treated animals, untreated (2.3‐fold) controls and mice immunized with DNA (1.4‐fold) demonstrated weaker increases in the number of CFU‐E colonies at day 40 (Fig. 4C). The relative percentage of CFU‐E in mice treated with MOG was significantly increased at 6 days from 4.6 to 21% (4.6‐fold) and then gradually decreased to 10.9% at day 40. In the case of non‐immunized and DNA‐treated mice, very weak changes in the average relative percentage of CFU‐E colonies from 0 to 40 days (from 4.6 to 6 and to 5.4%, respectively) were observed (Fig. 5B).

The total number of CFU‐GM colonies of untreated mice gradually increased from zero to 40 days by a factor of ~1.4‐fold (Fig. 4D), while their relative content (%) was increased at 6‐9 days and then it was slowly decreased to 40 days (Fig. 5C). The curves corresponding to the changes in total number and relative per cent of CFU‐GM in the case of DNA‐treated and untreated mice were very similar (Fig 4D and C). The relative per cent of CFU‐GM of mice treated with MOG was generally decreased from 0 to 40 days with a marked uptick at 18–20 days.

The total number of CFU‐GEMM colonies in the case of untreated mice gradually decreased and at 40 days was ~2.1‐fold lower than that at zero time (Fig. 4E). The treatment of mice with MOG led to a significant decrease in the total number of CFU‐GEMM compared with that for untreated mice, and at 18 days, this value was 6.6‐fold lower than at the beginning of the experiment (Fig. 4E). Immunization of mice with DNA led a nearly opposite effect; at 24 days, the total number of CFU‐GEMM was 1.6 higher than at time‐point zero. The curves corresponding to the changes in the total number and percentage content for CFU‐GEMM have a very similar time course (Figs 4E and 5D). Thus, it is obvious that the differentiation profiles of bone marrow stem cells are very different for all groups of mice analysed. These differences are especially pronounced in the case of immunization of mice with MOG and DNA. In the case of CFU‐E, CFU‐GM and CFU‐GEMM colonies, they show the opposite behaviour (Fig. 5).

### Lymphocyte proliferation in different mouse organs

**Figure 6:**
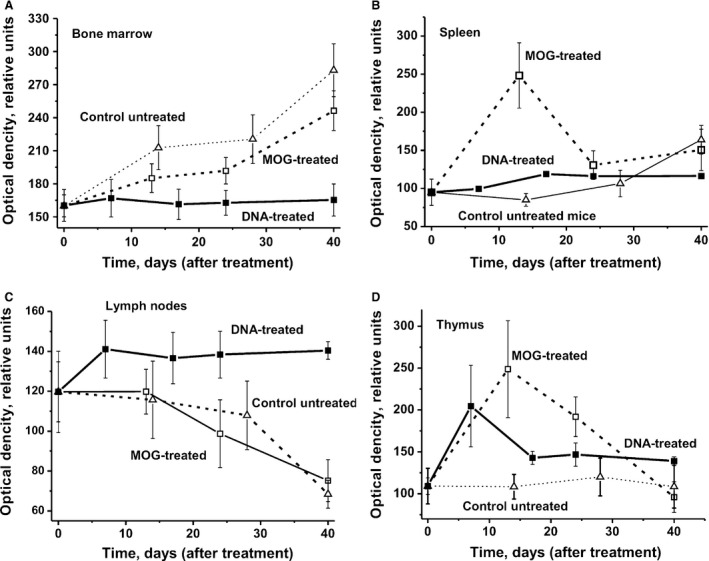
Changes over time in the optical density corresponding to the relative level of lymphocyte proliferation in bone marrow (**A**), spleen (**B**), lymph nodes (**C**) and thymus (**D**) in the case of untreated, MOG‐ and DNA‐treated mice. The error in the optical density determination from three independent experiments in the case of every mouse of each group did not exceed 7–10%. For other details, see Materials and methods.

As shown earlier, production of autoantibodies and abzymes is associated with the change in the differentiation profiles of HSCs and an increase in the level of lymphocyte proliferation (sum of T and B cells) in bone marrow and other organs during spontaneous development of autoimmune diseases as well as acceleration of them by immunization of MRL‐lpr/lpr mice with DNA 33, 34, 35 and EAE mice with MOG 37. Interestingly, the differentiation profile of HSCs following treatment of mice with DNA is different compared to that in the case of untreated or MOG‐induced development of the disease (Fig. 5). The relative number of different colony units that are produced after immunization of mice with DNA also changed very weakly over time (Fig. 4A). The increase in hydrolysis of MOG and MBP associated with the immunization of mice with DNA is relatively weak, and an increase in DNase activity is observed only in a very late time‐point (40 days) (Fig. 2). All these data agree very well with the fact that immunization of mice with DNA in contrast to untreated mice and MOG‐induced development of EAE does not lead to a noticeable increase in the proliferation level of lymphocytes in the bone marrow (Fig. 6A).

It was shown earlier that IgGs from the sera of healthy 2‐ to 3‐month‐old autoimmune‐prone MRL‐lpr/lpr mice are catalytically inactive 33, 34, 35. Immunization of these mice with DNA results in significant increase in abzyme activities and proteinuria, although it does not have a marked effect on the differentiation profile of HSCs and is mostly associated with a significant increase in the level of proliferation in lymphocytes in some mouse organs (thymus, spleen and lymph nodes) 35. A similar situation was observed after immunization of C57BL/6 mice with DNA (Fig. 6). Immunization with DNA led to a marked or significant increase in the level of proliferation in lymphocytes only from the spleen, lymph nodes and the thymus. Interestingly, the increase in the level of proliferation in lymphocytes after treating mice with DNA was lower for the spleen (Fig. 6B), comparable for the thymus (Fig. 6D), but higher for lymph nodes (Fig. 6C) compared with animals immunized with MOG.

Overall, immunization of mice with MOG and DNA leads to the production of abzymes in their bone marrow. In this regard, it should be noted that the relative activities of abzymes hydrolysing MBP, DNA and oligosaccharides in the bone marrow of the same MS patients are about 30–60 times higher than in their blood 25, 26, 28. This increase in abzyme activities indicates a change in the differentiation profile of lymphocytes in the bone marrow, leading to the appearance of cells producing abzymes.

From this point of view, changes in the levels of lymphocyte proliferation in different organs of C57BL/6 and MRL‐lpr/lpr mice show similar behaviour. At the same time, immunization of MRL‐lpr/lpr mice with DNA led to a strong increase in abzyme activities and proteinuria 35, while no increase in these indices were observed for C57BL/6 mice except an enhancement of DNase activity at day 40 (Figs 1 and 3).

### Cell apoptosis assay

**Figure 7:**
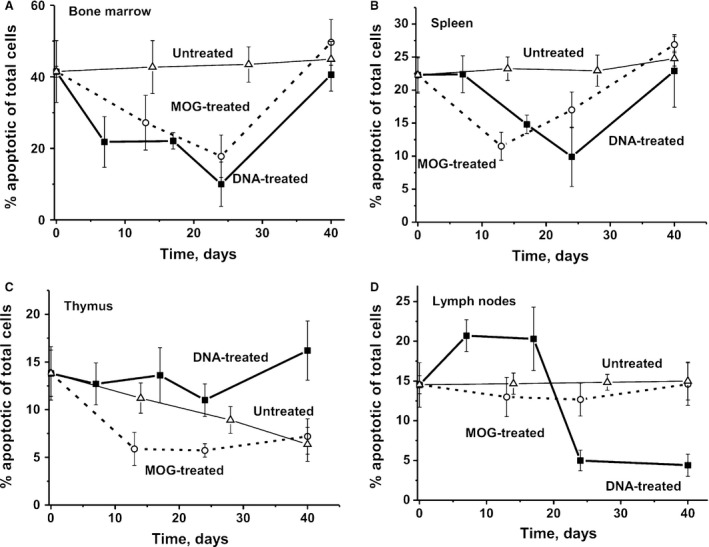
Changes in the relative level of apoptosis (%) in bone marrow **(A),** spleen (**B**), lymph nodes (**C**) and thymus (**D**) for untreated, DNA‐ and MOG‐treated mice. The error in the estimation of apoptosis from three independent experiments in the case of every mouse of each group did not exceed 7–10%. For other details, see Materials and methods.

Untreated mice showed a very slow increase in apoptosis over 40 days in all organs except the thymus, where it gradually decreased (Fig. 7). A comparable reduction in apoptosis in the case of bone marrow and the spleen was observed at 10‐25 days after treatment of mice with DNA and MOG (Fig 7A and B). At the same time, after treatment of mice with DNA, there was no significant change in apoptosis levels over time in the thymus compared with a significant reduction in MOG‐treated mice (Fig. 7C). MOG treatment has no considerable effect on the level of apoptosis in the case of lymph nodes. However, while immunization of mice with DNA resulted in a strong increase in apoptosis between days 7 and 17, a dramatic reduction followed up to day 40 (Fig. 7D). Thus, MOG and DNA show the similar effects on the level of apoptosis in the case of bone marrow and spleen, but completely different effects in the case of thymus and lymph nodes.

It was shown previously that the level of cell apoptosis in the case of bone marrow of control, untreated, non‐autoimmune CBA mice (16.8%) is approximately 2.5‐fold lower than that for EAE mice (41.6%) 37. The defects of immune system of EAE mice begin with specific violations in the bone marrow, and even at 3 months of age, these mice demonstrate well detectable catalytic activities of abzymes. Therefore, one can exclude that increased level of apoptosis in bone marrow of EAE mice may be a consequence of a developing of some autoimmune processes in the bone marrow of these mice even in 3 months of age.

## Discussion

C57BL/6 mice are used as a model autoimmune‐prone strain and develop EAE symptoms after treatment with MOG 36. It was shown previously that the appearance of antibodies exhibiting DNase and proteolytic activities in human and animal blood may be considered as a clear indicator of the onset of autoimmune reactions, which are typical for many autoimmune pathologies 10, 11, 12, 13, 14. At the same time, we have revealed highly detectable DNA‐, MBP‐ and MOG‐hydrolysing activities of IgGs in 3‐month‐old C57BL/6 mice 37. In addition, a significant increase in proteinuria, anti‐MOG‐, anti‐MBP‐ and anti‐DNA antibodies was observed for untreated C57BL/6 mice during days 0‐40 of the experiment 37. These findings suggest that C57BL/6 mice represent an autoimmune‐prone strain that is potentially capable of developing spontaneous autoimmune processes. The treatment of mice with MOG led to a significant increase in all enzymatic activities at stages corresponding to the beginning (6–8 days) and the acute phase of EAE (14–22 days) 37.

It was shown that development of SLE in MRL‐lpr/lpr mice and changes in EAE‐like parameters in C57BL/6 mice occur spontaneously and may be accelerated by immunization of mice with DNA 33, 34, 35 or with MOG 37, respectively. We recently compared changes in the differentiation profiles of bone marrow HSCs of MRL‐lpr/lpr and C57BL/6 mice 37. It was shown that the spontaneous development of SLE in MRL‐lpr/lpr mice and changes in EAE‐like parameters in C57BL/6 mice, as well as their acceleration after treatment with specific antigens (DNA and MOG, respectively), are characterized by very similar changes in the differentiation profiles of HSCs 37. Taking this into account, it was interesting to analyse what would happen to the differentiation profile of HSCs along with other autoimmune indexes after immunization of EAE mice with DNA as was performed previously with SLE mice. Overall, the results were surprising. In contrast to spontaneous and MOG‐induced changes, immunization of mice with DNA led to the absence of noticeable increases in proteinuria, a decrease in the concentrations of Abs to MOG and to DNA, as well as a reduction in the production of abzymes catalysing hydrolysis of MOG, MBP and DNA (Figs 1, 2 and 3). Treatment of EAE mice with MOG led to significant increase in all these indexes from days 6–7 to days 18–20, whereas immunization with DNA produced only a weak rise at day 40 (Figs 1, 2 and 3). After 40 days, immunization of mice with DNA only significantly increased the DNase activity of IgGs. This activity becomes 122‐fold higher than at time‐point zero and fivefold higher than the maximal activity seen after treatment with MOG. These data indicate that, in the case of EAE development in mice, DNA injection may prevent MOG‐induced alterations to some extent. One cannot exclude that C57BL/6 mice could generate a quick immune response only in the case of MOG and possibly MBP. In case of immunization with DNA, there seems to be a lag period that is needed for rearrangement of the immune system following the response to DNA.

Our results indicate that EAE in contrast to MRL‐lpr/lpr mice predisposed mainly to the EAE but not to SLE pathology, and the outset of the EAE is most probably may be associated with mice immunization only with MBP or its specific peptides. It is known that people can have a genetic predisposition to MS 1, 42, 43, 44 or to SLE disease 1. One cannot exclude, that similar to mouse models of EAE or SLE, some people genetically predisposed to MS can develop outset of multiple sclerosis only after their autoimmunization by MBP and products of its hydrolysis, while predisposed to SLE pathology only after immunization with DNA. However, it was shown that in the later stages of these pathologies, the blood of patients with MS and SLE contains abzymes with several different catalytic activities hydrolysing MBP, DNA, RNA, nucleotides and polysaccharides 9, 10, 11, 12, 13, 14. After immunization of EAE mice by DNA after 40 days of the experiment, the effective production of anti‐DNA antibodies with high DNase activity is observed (Fig. 3C). In other words, it cannot be excluded that people having genetic predisposition to the development of the MS cannot get in some condition the SLE or other autoimmune pathologies. Potentially, this can be related to different living conditions and other factors including the kind of auto‐ or foreign antigen in high concentrations affecting on various persons for a long time. For example, in each MS patient, the ‘relative stability’ of different organs and their functions to the destructive effect of transient immune system errors can be significantly different depending on the genetic background and environmental stress factors, including geographic ones 1, 45. In individual MS patients, the development of autoimmune reactions can be stimulated by different viral or bacterial infections as well as various toxic chemicals. Some indicators of diseases common for SLE and MS including rheological disturbances were observed 1. Antibody‐mediated neural cell injury and rheological disturbances in SLE represent the two principal suggested mechanisms of tissue injury in SLE patients 1. An interplay between these processes, underlying genetic factors, their modification by hormones, complicated by a number of secondary factors, may explain the wide spectrum of features encountered in this disease 1. Interestingly, there are examples of identical twins with a genetic predisposition to MS getting sick at about the same time, but one with multiple sclerosis, and other with SLE 45. Thus, it is possible to propose that the development of autoimmune processes in the EAE mice after their immunization with DNA (more similar to those in the case of SLE) and delay in the development of autoimmune processes comparing to that in the case of mice treatment with MOG may reflect the non‐universality of the genetic predisposition of these mice only to MS. One cannot exclude that a similar situation could be in case of some people genetically predisposed to MS or to SLE.

Taken together, autoimmune‐prone MRL‐lpr/lpr and C57BL/6 mice are characterized by the spontaneous development of a condition that is susceptible to autoimmune attacks. This is associated with significant and in many respects similar changes in the differentiation profile of bone marrow HSCs, differentiation and proliferation in lymphocytes of different organs including bone marrow, and the production of abzymes hydrolysing DNA. The development of other different autoimmune diseases is also probably associated with significant changes in the differentiation profile of HSCs and the differentiation and proliferation of lymphocytes, which may be an important prime cause for the initial stages of these autoimmune processes.

## Conflict of interest

The authors declare no conflicts of interest.
